# A Web-Based Mental Health Intervention to Improve Social and Occupational Functioning in Adults With Type 2 Diabetes (The Springboard Trial): 12-Month Outcomes of a Randomized Controlled Trial

**DOI:** 10.2196/16729

**Published:** 2020-12-01

**Authors:** Peter Andrew Baldwin, Samineh Sanatkar, Janine Clarke, Susan Fletcher, Jane Gunn, Kay Wilhelm, Lesley Campbell, Nicholas Zwar, Mark Harris, Helen Lapsley, Dusan Hadzi-Pavlovic, Helen Christensen, Judy Proudfoot

**Affiliations:** 1 Black Dog Institute Randwick Australia; 2 University of Melbourne Melbourne Australia; 3 UNSW Sydney Randwick Australia

**Keywords:** type 2 diabetes, depression, internet

## Abstract

**Background:**

People with type 2 diabetes mellitus (T2DM) often experience mental health symptoms that exacerbate illness and increase mortality risk. Access to psychological support is low in people with T2DM. Detection of depression is variable in primary care and can be further hampered by mental health stigma. Electronic mental health (eMH) programs may provide an accessible, private, nonstigmatizing mental health solution for this group.

**Objective:**

This study aims to evaluate the efficacy over 12 months of follow-up of an eMH program (myCompass) for improving social and occupational functioning in a community sample of people with T2DM and self-reported mild-to-moderate depressive symptoms. myCompass is a fully automated and self-guided web-based public health program for people with depression or anxiety. The effects of myCompass on depressive symptoms, diabetes-related distress, anxiety symptoms, and self-care behavior were also examined.

**Methods:**

Adults with T2DM and mild-to-moderate depressive symptoms (N=780) were recruited via online advertisements, community organizations, and general practices. Screening, consent, and self-report questionnaires were administered online. Eligible participants were randomized to receive either myCompass (n=391) or an attention control generic health literacy program (Healthy Lifestyles; n=379) for 8 weeks. At baseline and at 3, 6, and 12 months postintervention, participants completed the Work and Social Adjustment Scale, the Patient Health Questionnaire-9 item, the Diabetes Distress Scale, the Generalized Anxiety Disorder Questionnaire-7 item, and items from the Self-Management Profile for Type 2 Diabetes. Glycosylated hemoglobin measurements were obtained at baseline and 6 and 12 months postintervention.

**Results:**

A total of 38.9% (304/780) of the trial participants completed all postintervention assessments. myCompass users logged in on an average of 6 times and completed an average of 0.29 modules. Healthy Lifestyles users logged in on an average of 4 times and completed an average of 1.37 modules. At baseline, the mean scores on several outcome measures, including the primary outcome of work and social functioning, were close to the normal range, despite a varied and extensive recruitment process. Intention-to-treat analyses revealed slightly greater improvement at 12 months in work and social functioning for the Healthy Lifestyles group relative to the myCompass group. All participants reported equivalent improvements in depression anxiety, diabetes distress, diabetes self-management, and glycemic control across the trial.

**Conclusions:**

The Healthy Lifestyles group reported higher ratings of social and occupational functioning than the myCompass group, but no differences were observed for any secondary outcome. Although these findings should be interpreted in light of the near-floor symptom scores at baseline, the trial yields important insights into how people with T2DM might be engaged in eMH programs and the challenges of focusing specifically on mental health. Several avenues emerge for continued investigation into how best to deal with the growing mental health burden in adults with T2DM.

**Trial Registration:**

Australian New Zealand Clinical Trials Registry Number (ACTRN) 12615000931572; https://www.anzctr.org.au/Trial/Registration/TrialReview.aspx?id=368109&isReview=true

## Introduction

### Background

Type 2 diabetes mellitus (T2DM) affects over 1 million Australians [1] and increases the risk of psychiatric and neurodegenerative disorders [2,3]. Up to 40% of people with T2DM experience depressive symptoms [4,5], which appear to worsen the physical health via impaired psychosocial functioning, poorer self-care, and increased need for outpatient and inpatient health services [2,4,5]. The relationship between T2DM and depressive symptoms seems to be bidirectional [6], with one condition intensifying symptoms in the other [4]. Therefore, population-based mental health programs for depression in people with T2DM have the potential to reduce the substantial personal burden and public health impacts of these comorbid conditions.

Cognitive behavioral therapy (CBT) is the most established psychological treatment for depression. In people with T2DM, face-to-face CBT appears to be effective in improving not only depressive symptoms but also fasting glucose levels [7], self-care behaviors, and overall quality of life [8]. Although most T2DM management takes place in primary care, detecting depression in people with T2DM can be challenging, and it may not be feasible for general practitioners (GPs) to deal adequately with both mental health and diabetes care within a single consultation. Concerns about mental health stigma, treatment cost, and *clinician fatigue* can lead some patients to avoid seeking help [8]. The high rates of comorbid T2DM and depressive symptoms [9] also mean that already-stretched local health systems may struggle to provide services to every person needing help [10,11]. Scalable methods of delivering evidence-based psychological therapies may provide an answer to many of these challenges [7].

Electronic mental health (eMH) programs can be clinically effective and cost-efficient tools for increasing the availability of mental health services [12,13]. In previous trials, eMH programs have been effective in addressing both depressive symptoms and diabetes-related distress in people with T2DM, using both diabetes-specific content [14] or existing depression treatments that incorporate therapist assistance [15]. Importantly, data suggest that eMH interventions are most effective in the mild-to-moderate depressive symptom range [16] prevalent in T2DM [17,18]. Therefore, low-intensity, population-based eMH programs seem well suited to people with T2DM who are also experiencing problems with low mood.

myCompass is an eMH program for depression and anxiety, which, in contrast to the eMH programs described earlier, operates as a self-help program without therapist assistance or diabetes content. A large randomized controlled trial (RCT) demonstrated that myCompass users with mild-to-moderate depression and anxiety experienced a significant improvement in symptoms and functioning, compared with placebo [19]. Data from a feasibility study indicated that myCompass has the potential to have a positive impact on the functioning and depressive symptoms in people with diabetes [20]. Therefore, the primary aim of the SpringboarD trial was to evaluate the impact of myCompass on work and social functioning in adults with T2DM and mild-to-moderate depressive symptoms relative to a generic health literacy program. In our primary analysis at 3-month postintervention, both groups reported significant improvements, but there was no specific benefit of myCompass [21].

### Objectives

Further to our analysis of 3-month outcomes [21], the aim of this study was to establish if allocation to the myCompass intervention resulted in improvement of daily functioning of adults with T2DM and mild-to-moderate depressive symptoms across a 12-month period. We hypothesized that the participants using myCompass would report improvements in self-reported work and social functioning relative to the participants using a placebo health literacy program at the 6-month and 12-month follow-up. In addition to examining long-term outcomes from our trial (RCT), this study also explored long-term changes in health and clinical outcomes across the 12-month period of the trial and included a biological marker of glycemic control (glycosylated hemoglobin, HbA_1c_). Inclusion of a wide range of variables in this study enabled us to identify which psychosocial factors most affected change in both physical and mental health outcomes across the trial.

## Methods

### Design

This paper is a secondary analysis of a two-arm RCT called *SpringboarD*. The full SpringboarD trial protocol is detailed elsewhere [22]. Across the trial, outcomes were assessed at baseline and at 3-, 6-, and 12-month postrandomization. All the participants had uninterrupted access to usual diabetes treatment throughout the study. The study was approved by the Human Research Ethics Committee (HREC) at UNSW Sydney (HREC 15090) and registered with the Australia and New Zealand Clinical Trials Register (ACTRN12615000931572).

### Participants and Setting

Full details of recruitment for the SpringboarD trial are detailed in separate papers [21,23]. In summary, recruitment began in September 2015 and continued until November 2017. The trial was advertised via GPs in New South Wales and Victoria, professional associations (eg, the Australian Association of Practice Managers), print advertisements in national diabetes-related publications, and online (via Google and Facebook). Potential candidates were contacted via email through Black Dog Institute’s Volunteer Research Register and the Sax Institute’s *45 and Up Study*, a large, longitudinal cohort study of healthy aging described elsewhere [24]. All promotional materials directed potential candidates to a secure study-specific website, which guided interested participants through the consent process and provided instructions regarding completion of the screening questionnaires.

### Eligibility Criteria

Australian residents were eligible for SpringboarD if they were aged 18-75 years, diagnosed with T2DM by a medical doctor, scored ≥2 on the 2-item Patient Health Questionnaire [25] (indicating likely depression), and had access to an internet-connected device. People who scored ≥2 on the 2-item Patient Health Questionnaire proceeded to complete the Patient Health Questionnaire-9 (PHQ-9) [26] at screening to establish depressive symptom levels. People were excluded if they answered *no* to the question, *Are you able to read and write English easily?*; had extremely severe depressive symptoms on the full PHQ-9 (score > 19); had probable psychosis (measured by the psychosis screener developed for the Australian National Mental Health and Well-being Survey) [27]; were currently receiving face-to-face therapy for depression; had a recent (within 2 months) change in antidepressant medication; had an elevated suicide risk (assessed by item 9 of the PHQ-9); or had used the myCompass program previously. Participants ineligible due to severe depressive symptoms, elevated suicide risk, or probable psychosis were referred to professional mental health services.

### Randomization

Participants were allocated to the intervention and control conditions using computerized block randomization at a 1:1 allocation ratio, which was initiated automatically by the Black Dog Institute study management software after the completion of the baseline questionnaires. Allocation was concealed from participants and researchers.

### Interventions

#### Active Intervention (myCompass)

**Figure 1:**
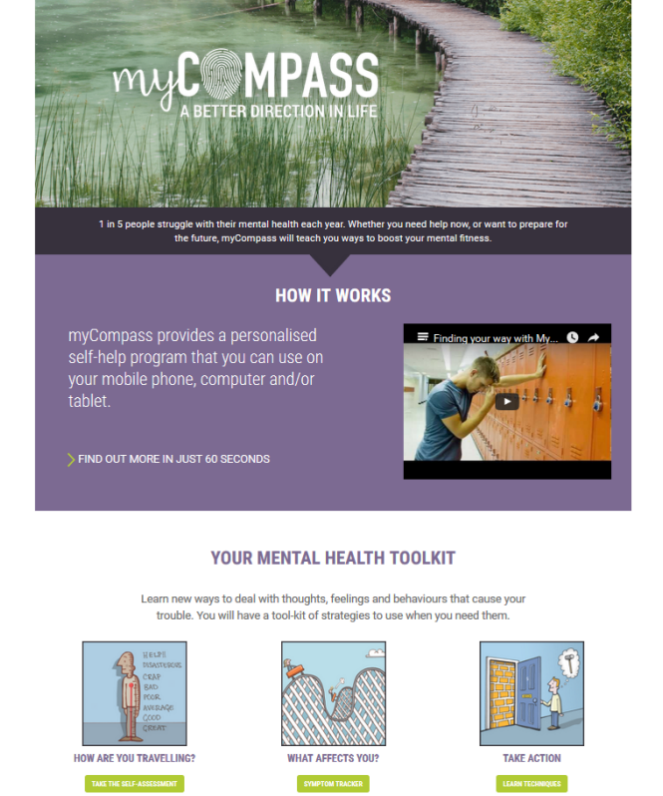
Screenshot of the myCompass landing page.

**Figure 2:**
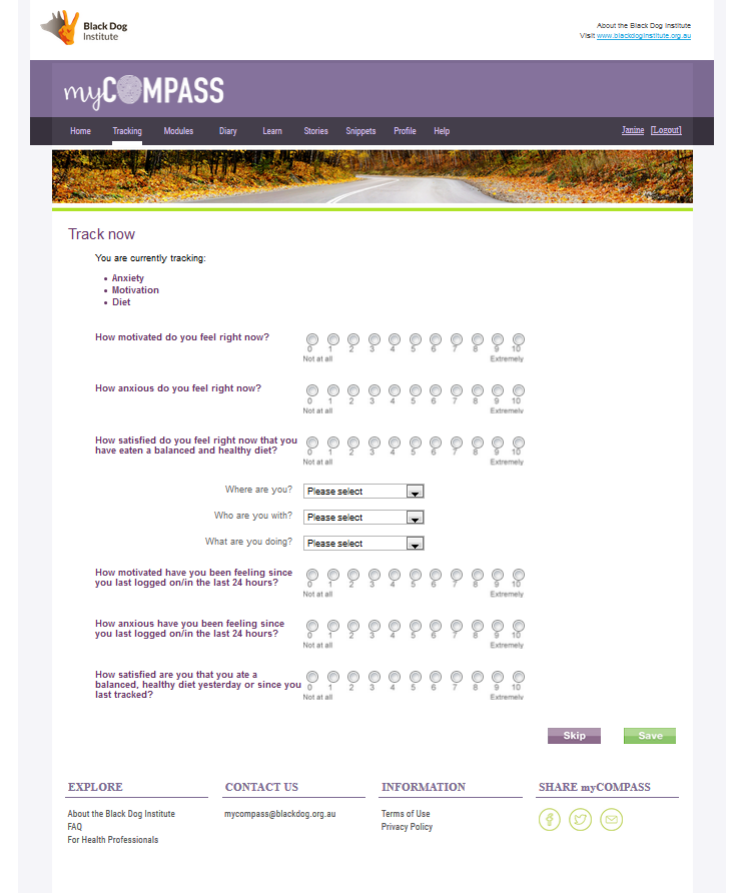
Screenshot of the myCompass self-monitoring page.

myCompass (Figure 1) is a fully automated eMH program [19] that contains 12 interactive mental health modules and allows users to self-monitor 3 of the total of 20 cognitive behavioral variables, such as eating, mood, or anxiety (Figure 2). Users can freely select modules and self-monitoring variables or opt for algorithm-based online guidance based on self-reported mental health symptoms given at registration or during program use. myCompass also provides SMS and/or email reminders, home practice activities, mental health care tips, motivational statements, and graphical reporting of self-monitoring data.

Participants randomized to the myCompass condition had full program access for 8 weeks, followed by a 4-week tailing-off period in which only the self-monitoring function was available. The program recommended that users complete 3 mental health modules and self-monitor up to 3 cognitive behavioral variables daily. myCompass users also received automated feedback via email about their program use in weeks 1, 3, 5, and 7.

myCompass user privacy is maintained via a password-protected login. All data are encrypted during transmission and stored on secure servers rather than on the user’s device. Participants’ myCompass user data were identified using the email address provided during the study registration. Once extracted from the myCompass server, data were de-identified and stored in a password-protected file.

#### Attention Control Intervention (Healthy Lifestyles)

The attention control program, *Healthy Lifestyles*, was adapted from a previous attention control program [28] to replicate the online, module-based structure of myCompass, without delivering any active CBT content. The 12 Healthy Lifestyles modules provide lifestyle information and interactive activities across 8 topics: (1) eye care, (2) skin care, (3) mental health, (4) home safety and comfort, (5) healthy interactions with digital devices, (6) safe driving, (7) travel planning, and (8) healthy eating. Participants in the Healthy Lifestyles condition received login reminders via email at weeks 1, 3, 5, and 7, but they did not receive feedback regarding their program use. To reflect the SMS functionality of myCompass, Healthy Lifestyles users also received a weekly SMS containing general health and well-being information for the first 4 weeks of the intervention period. Participants had full access to the Healthy Lifestyles program for 8 weeks.

### Outcome Measures

A summary of the measures obtained from participants at baseline and at 3-, 6-, and 12-month postrandomization is presented in Table 1.

**Table 1:** Measures obtained at each assessment phase.

Measures		Baseline	3 months	6 months	12 months
**Demographic and disease-related information**					
	Demographic data	✓^a^	—^b^	—	—
	Disease-relevant data	✓	✓	✓	✓
	Mental health history	✓	—	—	—
**Primary outcome**					
	WSAS^c^	✓	✓	✓	✓
**Secondary outcomes**					
	PHQ-9^d^	✓	✓	✓	✓
	DDS^e^	✓	✓	✓	✓
	GAD-7^f^	✓	✓	✓	✓
	SMP-T2D^g^	✓	✓	✓	✓
	HbA_1c_^h^	✓	—	✓	✓
	Days out of role	✓	✓	✓	✓
	Health service utilization	✓	✓	✓	✓

^a^Measurement taken.

^b^Measurement not taken.

^c^WSAS: Work and Social Adjustment Scale.

^d^PHQ-9: Patient Health Questionnaire-9.

^e^DDS: Diabetes Distress Scale.

^f^GAD-7: Generalized Anxiety Disorder Scale.

^g^SMP-T2D: Self-Management Profile for Type 2 Diabetes.

^h^HbA_1c_: glycosylated hemoglobin.

### Primary Outcome

The primary outcome for the trial was work and social functioning, measured by the Work and Social Adjustment Scale (WSAS). WSAS measures the daily functioning across 5 life domains: work, socializing, leisure, home, and personal relationships [29,30]. Scores range from 0 to 40, with higher scores indicating poorer functioning.

### Secondary Outcomes

Depressive symptoms were measured using the PHQ-9 scale [20], and anxiety symptoms were measured using the 7-item Generalized Anxiety Disorder Scale (GAD-7) [31]. Both scales are well validated and commonly used as outcome measures in chronically ill cohorts [32,33]. Each scale uses cutoff scores of 5, 10, and 15 to reflect mild, moderate, and moderately severe symptoms, respectively.

Diabetes distress—a person’s emotional adjustment to diabetes—was measured using the Diabetes Distress Scale (DDS) [34]. DDS is a 17-item questionnaire that provides an overall measure of diabetes distress along with 4 subscale scores that index (1) regimen-related distress, (2) interpersonal distress, (3) emotional burden of diabetes, and (4) distress related to interacting with health care providers. DDS total and subscale scores were calculated by averaging all items in the scale or subscale and ranged from 1 to 6, with higher scores indicating greater distress. A score of >3 indicates clinically significant distress.

Diabetes management was measured using a subset of items from the Self-Management Profile for Type 2 Diabetes (SMP-T2D). The SMP-T2D was developed to measure the level and perceived ease of engaging in common diabetes management behaviors, along with perceived coping, confidence dealing with diabetes, and ease of weight management [35]. As DDS also asks about coping and confidence in diabetes self-care, to reduce participant assessment burden, we administered only the SMP-T2D items that assess diabetes management behaviors across 4 domains: blood glucose monitoring, medication adherence, healthy eating, and exercise. Scores in each behavior domain are converted to a percentage of the previous week spent engaging in a particular diabetes management behavior. Higher scores indicate more time spent on diabetes management [35].

### Additional Measurements

We also collected baseline data regarding each participant’s diabetes history (eg, age of onset and treatment regimen), demographics (eg, age, gender, education, and occupation), and mental health history (eg, service use and previous diagnoses). With written consent, participants’ most recent HbA_1c_ results were provided by their GPs at baseline and 6- and 12-month follow-up. Service utilization for physical and mental health was captured at baseline and all follow-up points, as was days out of role, defined as the number of days in the previous 30 that participants were unable to perform work or normal activities because of problems with physical or mental health [36].

### Sample Size

Initial calculations identified that approximately 600 participants were required to detect a between-group effect of 0.3 in scores on the WSAS postintervention, with power of 80% and a two-tailed alpha level of .05, and a prediction of approximately 40% total attrition. Due to unexpectedly high attrition early in the trial, a further 180 participants were recruited to maintain sufficient power.

### Analyses

All mixed model repeated measure (MMRM) analyses were computed in *R* [37] using the Linear Models module in Jamovi v0.9 [38]. Model parameters were estimated using restricted maximum likelihood estimation, and error degrees of freedom were calculated using the Satterthwaite approximation. Repeated measures (level 1) were nested within individual levels (level 2), and a random intercept was fitted at the individual level to account for intra-individual correlations on repeated measures.

## Results

### Data Preparation

All data were inspected for outliers, skew, and kurtosis. Data from the Medication Adherence and Exercise domains for the SMP-T2D were highly left-skewed and kurtotic. Therefore, these data were not included in subsequent analyses. All regression coefficients were reported in unstandardized format and, therefore, gave an estimate of the effect size in dependent-variable units. For example, our estimate of the effect of age on PHQ-9 scores over time was –0.089, indicating that each year of increased age was associated with a reduction in PHQ-9 of 0.089 points.

### Sample Characteristics

**Figure 3:**
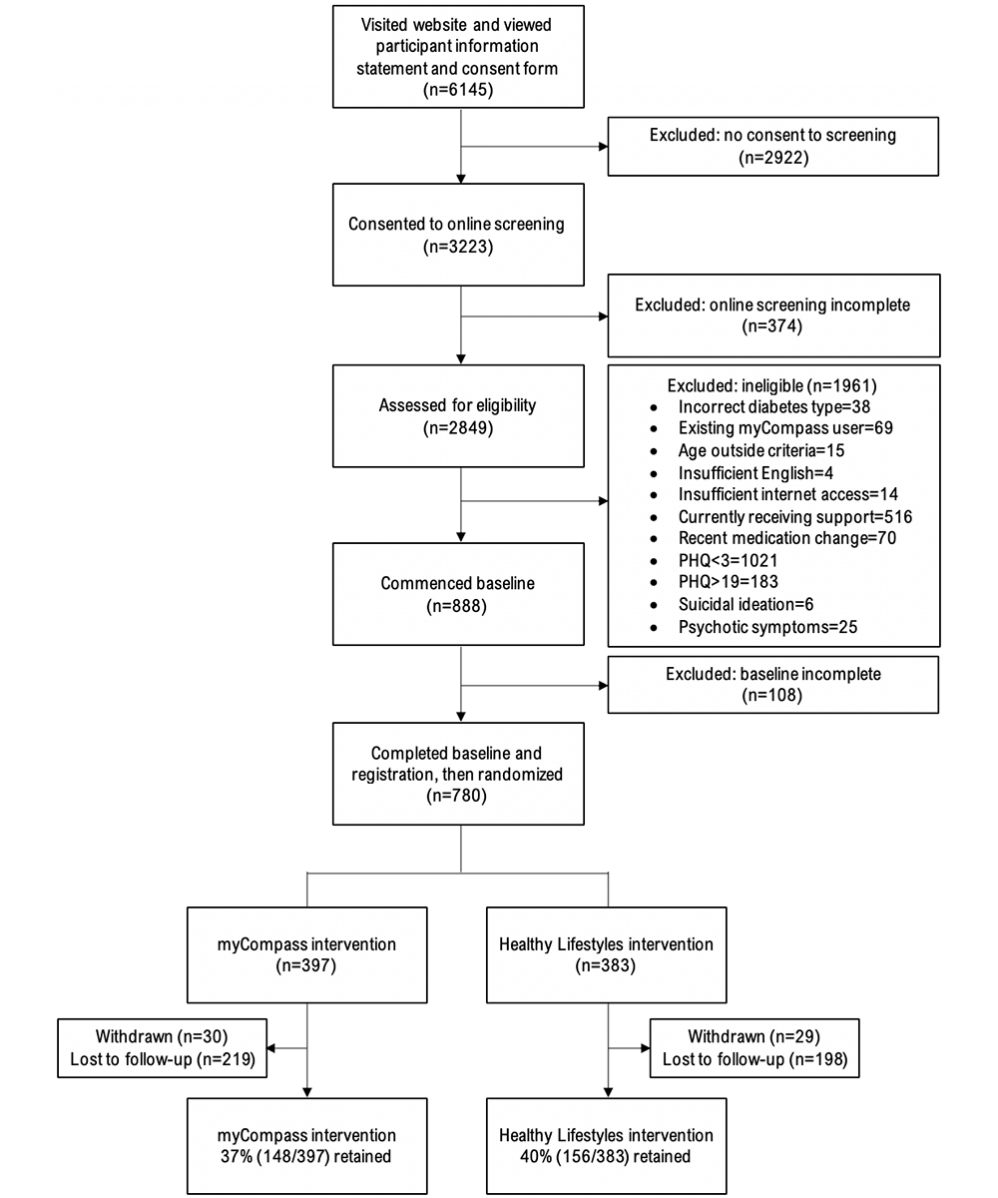
CONSORT (Consolidated Standards of Reporting Trials) participant flow diagram through the SpringboarD trial. PHQ: Patient Health Questionnaire-9.

Sample characteristics are presented in Table 2, and CONSORT (Consolidated Standards of Reporting Trials) metrics are shown in Figure 3. A total of 52.49% (3223/6145) of the individuals who visited the study website consented to online screening, and 27.55% (888/3223) of those participants were eligible to proceed to the baseline assessment. The most common reasons for ineligibility were the absence of depressive symptoms, that is, a score of <2 on the PHQ-2 (1021/1961, 52.1%), current face-to-face mental health treatment (516/1961, 26.31%), and severe depressive symptoms (183/1961, 9.3%). In total, 87.8% (780/888) of eligible individuals completed the baseline measures and were randomized and 7.3% (57/780) subsequently withdrew consent, leaving a final sample of 81.4% (723/888) of the eligible participants.

Table 2 presents the intervention and control group participants’ characteristics at baseline. Participants were, on average, aged 58 years (SDs were 10.6 for intervention group participants and 10.0 for control group participants) and were mostly female (437/723, 60.4%). Randomization successfully matched the groups based on demographic characteristics as well as mental health– and diabetes-related histories. Participants allocated to the intervention group reported taking antidepressants for slightly longer (mean 98 days, SD 95 days) than the control group participants (mean 74 days, SD 56 days), and there were slightly more intervention participants who managed their diabetes with diet (230/368, 62.5%) than control group participants (207/355, 58.3%).

**Table 2:** Baseline means (SDs) for myCompass and Healthy Lifestyles Groups.

Characteristics				Percentage, n (%); (N=723)		myCompass (n=368), mean (SD)		Healthy lifestyles (n=355), mean (SD)		*P* value
**Demographics**										N/A^a^
	Age (years)			N/A		57.7 (10.6)		57.7 (10.0)		
	Female			465 (64.3)		229 (62)		236 (66)		
	Married			387 (53.5)		204 (55)		183 (52)		
	Employed			351 (48.5)		173 (47)		178 (50)		
**Education level**										N/A
	Secondary school or lower			220 (30.4)		112 (30)		108 (30)		
	Trade certificate or diploma			270 (37.3)		133 (36)		137 (39)		
	University undergraduate or more			233 (32.2)		123 (33)		110 (31)		
**Mental health**										N/A
	**Lifetime history**									
		Sought professional support for mental health	571 (78.9)		296 (80)		275 (77)			
		Received mental health diagnosis	300 (41.5)		155 (42)		145 (41)			
		Diagnosed with depressive symptoms or major depressive disorder	279 (38.6)		143 (38)		136 (38)			
	**Past 6 weeks**								N/A	
		Sought professional support for mental health	113 (15.6)		65 (18)		48 (14)			
	**Current**									
		Taking antidepressant medication	241 (33.3)		125 (34)		116 (33)		N/A	
		Months using antidepressant medication	N/A		97.70 (94.72)		73.67 (56.21)^b^		.04	
**Diabetes**										
	Age at diagnosis (years)			N/A		46.6 (11.1)		47.2 (10.9)		N/A
	**Diabetes treatment**									
		Healthy eating	437 (60.4)		230 (63)		207 (58)^b^		.04	
		Physical activity	323 (44.7)		176 (48)		147 (41)		N/A	
		Oral medication	583 (80.6)		295 (80)		288 (81)		N/A	
		Insulin	216 (29.9)		113 (31)		103 (29)		N/A	
		Exenatide	32 (4.4)		21 (6)		11 (3)		N/A	
**Past 6 weeks**										N/A
		Visited general practitioner for diabetes	419 (57.9)		218 (59)		201 (57)			
		Frequency of general practitioner visit	N/A		1.31 (.78)		1.37 (.71)			
		Hospitalized for diabetes	24 (3.3)		13 (4)		11 (3)			
		Frequency of hospitalization for diabetes	N/A		1.46 (1.5)		1.36 (.9)			

^a^N/A: not applicable.

^b^Means differ significantly at *P*<.05

### Intention-to-Treat Analyses (MMRM)

The estimated marginal means for all study outcomes are presented in Table 3, and the fixed effects from the MMRM analyses are given in Table 4. WSAS showed a significant improvement between pre- and postintervention, which was maintained at both 6- and 12-month follow-up. The effects of time differed significantly across the groups, with the Healthy Lifestyles group unexpectedly showing a greater improvement in work and social functioning across the trial than the myCompass group.

No other between-group differences were observed. All participants showed small but significant improvements on PHQ-9, DDS, and GAD-7 between baseline and postintervention, and these improvements were maintained across both follow-up periods. Similarly, all participants maintained significant increases in the healthy eating and blood glucose monitoring domains of the SMP-T2D across the trial, irrespective of group. HbA_1c_ decreased significantly between baseline and 6 months for all participants, but the reduction from baseline was no longer significant at 12 months.

**Table 3:** Estimated marginal means and SEs on key outcome variables for the myCompass and Healthy Lifestyle groups.

Variables and groups		Baseline, MC^a^ (n=368), HL^b^ (n=355), mean (SE)		3 months, MC (n=232), HL (n=241), mean (SE)		6 months, MC (n=216), HL (n=221), mean (SE)		12 months, MC (n=148), HL (n=156), mean (SE)	
**WSAS^c^**									
	HL		12.157 (0.456)		10.827 (0.523)		10.972 (0.538)		11.214 (0.542)
	MC		13.440 (0.453)		12.055 (0.530)		11.926 (0.541)		11.642 (0.558)
**PHQ-9^d^**									
	HL		10.802 (0.260)		8.411 (0.302)		8.379 (0.311)		8.017 (0.314)
	MC		11.199 (0.258)		8.814 (0.307)		8.269 (0.314)		8.426 (0.325)
**GAD-7^e^**									
	HL		7.236 (0.230)		6.231 (0.267)		6.322 (0.276)		5.929 (0.306)
	MC		7.490 (0.228)		6.755 (0.271)		6.278 (0.277)		5.949 (0.317)
**DDS^f^**									
	HL		2.536 (0.048)		2.237 (0.054)		2.205 (0.055)		2.203 (0.055)
	MC		2.498 (0.047)		2.275 (0.054)		2.146 (0.055)		2.199 (0.057)
**SMP-HE^g^**									
	HL		48.595 (1.522)		54.411 (1.742)		52.245 (1.788)		55.550 (1.804)
	MC		50.909 (1.510)		53.206 (1.761)		55.053 (1.800)		52.951 (1.854)
**SMP-BG^h^**									
	HL		45.705 (2.075)		53.760 (2.386)		51.664 (2.451)		50.991 (2.478)
	MC		50.083 (2.058)		49.361 (2.425)		51.761 (2.473)		49.643 (2.545)
**HbA_1c_^i^**									
	HL		7.465 (0.089)		N/A^j^		7.255 (0.100)		7.256 (0.106)
	MC		7.527 (0.088)		N/A		7.400 (0.103)		7.534 (0.109)

^a^MC: myCompass.

^b^HL: Healthy Lifestyles.

^c^WSAS: Work and Social Adjustment Scale.

^d^PHQ-9: Patient Health Questionnaire-9.

^e^GAD-7: Generalized Anxiety Disorder Scale.

^f^DDS: Diabetes Distress Scale.

^g^SMP-HE: Self-Management Profile for Type 2 Diabetes-Healthy Eating.

^h^SMP-BG: Self-Management Profile for Type 2 Diabetes-Blood Glucose Monitoring.

^i^HbA_1c_: glycosylated hemoglobin (mmoL/L).

^j^N/A: not applicable.

**Table 4:** Mixed model repeated measures fixed effects for time, group, and time×group on primary and secondary outcome variables.

Variables and effects		*β* ^a^	SE	95% CI	*df*	*t* test	*P* value
**WSAS^b^**							
	Group	1.163	0.571	0.045 to 2.282	705.855	2.039	.04
	T3^c^	–1.408	0.360	–2.113 to –0.703	1452.986	–3.913	<.001
	T6^d^	–1.376	0.369	–2.100 to –0.653	1458.523	–3.729	<.001
	T12^e^	–1.380	0.377	–2.118 to –0.642	1459.629	–3.663	<.001
**PHQ-9^f^**							
	Group	.322	0.315	–0.295 to 0.939	716.640	1.022	.31
	T3	–2.430	0.221	–2.863 to –1.998	1491.246	–11.009	<.001
	T6	–2.703	0.226	–3.147 to –2.260	1498.607	–11.941	<.001
	T12	–2.811	0.231	–3.265 to –2.358	1500.770	–12.157	<.001
**GAD-7^g^**							
	Group	.223	0.281	–0.328 to 0.773	683.228	0.793	.43
	T3	–0.926	0.195	–1.309 to –0.544	1365.156	–4.751	<.001
	T6	–1.105	0.200	–1.498 to –0.712	1374.206	–5.513	<.001
	T12	–1.472	0.224	–1.912 to –1.033	1386.943	–6.566	<.001
**DDS^h^**							
	Group	–0.029	0.065	–0.157 to 0.099	692.095	–0.442	.66
	T3	–0.272	0.034	–0.339 to –0.205	1391.445	–7.953	<.001
	T6	–0.349	0.035	–0.418 to –0.280	1394.559	–9.935	<.001
	T12	–0.325	0.036	–0.396 to –0.255	1394.854	–9.046	<.001
**SMP-HE^i^**							
	Group	.944	1.913	–2.806 to 4.694	683.100	0.493	.62
	T3	4.357	1.182	2.040 to 6.673	1419.606	3.686	<.001
	T6	4.110	1.214	1.731 to 6.489	1424.907	3.386	<.001
	T12	4.811	1.239	2.383 to 7.240	1425.917	3.883	<.001
**SMP-BG^j^**							
	Group	.988	2.549	–4.009 to 5.984	692.279	0.387	.70
	T3	3.998	1.651	0.762 to 7.234	1435.328	2.422	.02
	T6	4.043	1.693	0.726 to 7.361	1441.001	2.389	.02
	T12	2.683	1.728	–0.703 to 6.070	1442.227	1.553	.12
**HbA_1c_^k^**							
	Group	.139	0.118	–0.092 to 0.370	484.986	1.176	.24
	T6	–0.169	0.068	–0.302 to –0.037	627.037	–2.507	.01
	T12	–0.113	0.072	–0.255 to 0.029	638.526	–1.561	.12

^a^*β* is the unstandardized regression coefficient for the effect holding constant age, sex, years since diabetes diagnosis, use of diabetes medication (yes/no), and use of psychiatric medication (yes/no).

^b^WSAS: Work and Social Adjustment Scale.

^c^T3: 3-month follow-up.

^d^T6: 6-month follow-up.

^e^T12: 12-month follow-up.

^f^PHQ-9: Patient Health Questionnaire-9.

^g^GAD-7: Generalized Anxiety Disorder Scale.

^h^DDS: Diabetes Distress Scale.

^i^SMP-HE: Self-Management Profile for Type 2 Diabetes-Healthy Eating.

^j^SMP-BG: Self-Management Profile for Type 2 Diabetes-Blood Glucose Monitoring.

^k^HbA_1c_: glycosylated hemoglobin (mmoL/L).

### Change-Over-Time Analyses

Small but significant improvements in the functioning and well-being of the participants were observed over the course of the study. To explore the contribution of psychosocial factors to these improvements, we reran the MMRM analyses without the grouping factor (thereby estimating change over time for the full cohort) for all variables that did not differ by group (ie, all secondary outcomes). Each model contained all psychosocial variables simultaneously, so each effect was estimated while holding all other covariates constant. The unique effects of psychosocial variables on improvements over time are presented in Table 5.

**Table 5:** Mixed model repeated measure fixed effects of covariates on change over time in secondary outcome variables.

Covariate	PHQ-9^a^		GAD-7^b^		DDS^c^		SMP-HE^d^		SMP-BG^e^		HbA_1c_^f^	
	*β* ^g^	SE	*β*	SE	*β*	SE	*β*	SE	*β*	SE	*β*	SE
Age	–0.089^h^	0.016	–0.101^h^	0.014	–0.035^h^	0.003	0.579^h^	0.098	0.487^h^	0.132	–0.044^h^	0.006
Sex	0.381^h^	0.326	0.063	0.290	0.161	0.064	–3.421^i^	1.989	–6.145^i^	2.683	–0.026	0.119
Psychiatric meds	1.177	0.305	0.326	0.272	0.011	0.060	–1.584	1.864	–0.492	2.514	0.053	0.111
T2DM^j^ meds	–0.178	0.388	0.017	0.346	0.007	0.076	–1.169	2.368	–6.488^i^	3.198	0.498^h^	0.142
Years since diagnosis	0.042^i^	0.020	0.017	0.018	0.012^h^	0.004	–0.136	0.123	–0.220	0.166	0.033^h^	0.007

^a^PHQ-9: Patient Health Questionnaire-9.

^b^GAD-7: Generalized Anxiety Disorder Scale.

^c^DDS: Diabetes Distress Scale.

^d^SMP-HE: Self-Management Profile for Type 2 Diabetes-Healthy Eating.

^e^SMP-BG: Self-Management Profile for Type 2 Diabetes-Blood Glucose Monitoring.

^f^HbA_1c_: glycosylated hemoglobin (mmoL/L).

^g^*β* is the unstandardized regression coefficient for the effect.

^h^*P*<.01

^i^*P*<.05

^j^T2DM: type 2 diabetes.

Several psychosocial factors have demonstrated significant contributions to changes in the functioning and well-being over time. Age was the most consistent contributor, with each additional year of age associated with lower depression, anxiety, and diabetes distress; better dietary control and blood glucose level (BGL) monitoring; and better glycemic management. Being female was associated with higher depression over time and less time spent eating healthy food or BGL monitoring. Being on medication for T2DM was associated with less self-reported BGL monitoring but better glycemic management. Each additional year of holding a diabetes diagnosis was associated with higher depression and diabetes distress and poorer glycemic management.

### Study Attrition

Of the total trial participants, 37.3% (148/397) of the participants from the myCompass program and 40.3% (156/387) of the participants from the Healthy Lifestyles program provided at least one measurement at the final time point (labeled *full completers*). To explore factors that affected engagement with the trial, we conducted a multivariate analysis of variance comparing noncompleters (participants who did not provide any postintervention measures) with full completers at baseline. Differences in diabetes distress and glycemic management were identified, with noncompleters reporting significantly more severe diabetes distress (*F*_1,783_=6.784; *P*=.009; *d*=0.30) and higher HbA_1c_ (*F*_1,783_=4.368; *P*=.04; *d*=0.24). Although these effects were small, it appears that diabetes distress and glycemic control were the main differentiating factors between those who completed the trial and those who did not.

### Program Use and Feedback

myCompass participants logged in on an average of 6 times (SD 9.01; range 1-71), started an average of 0.71 modules (SD 1.18; range 0-8), completed an average of 0.29 modules (SD 0.87; range 0-7), and monitored their symptoms an average of 2 times (SD 5.79; range 0-53). Healthy Lifestyles participants logged in on an average of 4 times (SD 3.22; range 1-17), started an average of 2.61 modules (SD 2.78; range 0-8), and completed an average of 1.37 modules (SD 2.24; range 0-8). There were no differences between participants who logged into their assigned program and those who did not, apart from a slightly higher GAD-7 score reported by myCompass users who logged in (*F*=10.76, *P*=.001; *d*=0.39). No measure of program engagement correlated with any baseline measurement. With respect to program acceptability, approximately 55% of myCompass participants and 11% of Healthy Lifestyles participants reported that their assigned program was convenient and easy to use.

## Discussion

### Principal Findings

This trial examined the efficacy of an unguided eMH program (myCompass) for improving work and social functioning in people with T2DM and mild-to-moderate depressive symptoms, relative to an attention control program (Healthy Lifestyles). Contrary to our hypothesis, the Healthy Lifestyles group showed significantly greater improvement in work and social functioning than the myCompass group across the 12-month trial period. Irrespective of the intervention, all study participants reported significant improvements in their mental health and diabetes management by the end of the trial.

In our primary analysis that examined differences at our first (3-month) follow-up period [21], the Healthy Lifestyles group also showed a slightly greater improvement in medication adherence. Both these effects are surprising, given that the Healthy Lifestyles program has been a reliably inert control in previous trials [28]. Taken together, these effects suggest that the Healthy Lifestyles control intervention comprised more active ingredients than we had intended or was presented in a way that was particularly engaging to our chronically ill sample. Perhaps reflecting on general health prompted small behavioral changes, which gradually improved the overall functioning. Alternatively, a broad health literacy program may have been less confronting than a mental health program, which may be experienced as stigmatizing and adding greater health burden. This was suggested in a small qualitative study we previously conducted, in which young people with type I diabetes mellitus and T2DM reported that a focus on mental health negatively influenced their decision to take up and engage in eMH services [39]. The findings of this study provide a useful starting point for future research that should include further qualitative investigations of how and why people with diabetes use online mental health support.

All participants reported significant reductions in depression, anxiety, and diabetes distress throughout the trial, regardless of group. Participants also reported increases in the time spent eating healthily and monitoring blood glucose, along with small improvements in glycemic management, as measured by HbA_1c_. These improvements over time did not meet the criteria for clinical significance but were robust across the sample and warrant consideration. It is an inescapable part of undertaking RCTs that require self-reported baseline measures that participants are assisted in reflecting on their own diabetes management, which can lead to improvements in diabetes health [38]. Being part of SpringboarD may have also created small increases in participants’ health literacy across the trial, leading to commensurate changes in diabetes management. As mentioned earlier, the effects were generally small and require replication before any firm conclusions are drawn.

The relationship between self-reported diabetes management behavior and glycemic management in our sample was interesting. Both groups reported improvements in healthy eating and blood glucose monitoring throughout the trial; however, the total time spent on each remained at around 50% of the prior week. Nonetheless, all participants maintained HbA_1c_ levels within the recommended range of 7%-8% [40] throughout the trial. This finding was unexpected and suggested that some self-management behaviors may be effective even when not applied consistently throughout the week. Future research to establish the levels of self-management that are both practicable and effective may be fruitful, given the importance of lifestyle management in diabetes care [40]. Of course, such research should also consider other relevant factors not included in our analyses, such as diabetes medication effectiveness and adherence.

This study extended from our primary analyses [21] by examining the contribution of psychosocial factors to changes in the functioning and well-being over 12 months in adults with T2DM and mild depressive symptoms. In line with recent Australian data [41], age was a robust contributing factor, with being older associated with lower anxiety, depression, and stress; better self-management; and lower HbA_1c_. Being female was associated with higher depression scores and poorer self-management. As may be expected, being on diabetes medication was associated with poorer BGL monitoring and higher HbA_1c_ likely related to longer duration and/or greater severity of disease.

Interestingly, having held a T2DM diagnosis for longer was associated with increased depression, diabetes distress, and HbA_1c_ levels, which may seem counterintuitive given that increased age was negatively associated with these factors. This also conflicts with previous data [42], indicating that both age and years since diagnosis predict poor glycemic management. Our findings suggest that although older people experience better functioning and well-being possibly related to health behaviors, living with T2DM longer is detrimental irrespective of age, possibly due to the worsening of cerebrovascular comorbidities over time [2]. Although recent data suggest that diabetes management may improve with age [41], the time postdiagnosis appears to be a separate and potentially detrimental factor in diabetes health. It may, therefore, be useful to consider these separately, both in future research and when assessing risk at the patient level.

Our lack of a treatment effect was surprising, given that myCompass showed promise as a treatment for depressive symptoms in a pilot trial of adults with diabetes [20], and myCompass has demonstrated efficacy in reducing depressive symptoms in the general community [19]. As discussed in our primary outcomes paper [21], this may be explained by methodological or population differences between previous work and this trial. Engagement in the myCompass program was lower than that observed in an earlier trial [19], giving rise to the possibility that people with diabetes may require or prefer more tailored interventions that directly address the challenges of diabetes management [41]. The lack of a treatment effect also likely reflects the impact of systematic attrition that resulted in near-floor mean baseline scores. Attrition is a well-known phenomenon in eMH research [43], and our data provide further impetus for an ongoing discussion of methodology in this area.

### Strengths and Limitations

Our challenges with recruitment and attrition, although not ideal, were ultimately informative. Participants with the highest symptoms of distress and impairment tended to leave the study. As a result, near-normal scores on baseline variables weakened tests of efficacy, and our results are largely indicative of people with T2DM and mild distress or impairment. Nonetheless, the inclusion of multiple follow-up assessments in this analyses and large sample that our study comprised afforded us the opportunity to extend the trial findings by analyzing recruitment strategies [23] and examining study attrition in both short and long terms.

At our first follow-up point, people who had left the study were characterized by more severe depressive and anxiety symptoms, greater diabetes-related distress, and poorer medication adherence [21]. However, by the final follow-up point, study-leavers were differentiated from study-completers by increased diabetes distress and poorer glycemic management. This suggests that in diabetes-related trials, short-term retention may be impacted by psychiatric factors (such as depression or anxiety), whereas diabetes-specific factors (such as emotional adjustment to diabetes) may impact both short- and long-term retention. This may reflect overall fatigue in chronic disease management.

Our insights could be valuable for future research. Retention strategies for future studies may need to vary across study phases, and the impact of this apparently biphasic pattern of attrition could be taken into account when analyzing results. In addition, future studies could continue this contribution to methodological improvements by further investigating factors influencing recruitment and trial engagement. For example, the association between age and improved functioning suggests that future recruitment strategies should focus on younger participants to ensure interventions are trialed with those who most need support and, therefore, are most likely to derive measurable benefits.

### Conclusions

The SpringboarD trial aimed to determine if a public health eMH program, myCompass, could improve the work and social functioning in adults with T2DM and mild-to-moderate depressive symptoms. The trial also sought to examine the impact of myCompass on a range of physical and mental health outcomes. There was a small, unexpected benefit to our control group in terms of work and social functioning, which suggests the need for future research to examine the value of generic health literacy tools. Neither control nor intervention programs were found to yield specific mental health benefits, but systematic attrition likely hampered true tests of efficacy by yielding only a mildly symptomatic sample.

Nonetheless, the trial itself revealed valuable insights into studying mental health in the context of T2DM. Early-stage attrition seems to be affected by mental health, whereas late-stage attrition seems more impacted by diabetes health. Increasing age appears to be associated with a gradual lift in both mental and physical health in T2DM; thus, research may show benefits from targeting younger cohorts. As mental health continues to be a significant contributor to morbidity in diabetes, increasingly refined approaches are required to meet the sizable demand for mental health support in people with T2DM.

## Abbreviations

BGL: blood glucose level
CBT: cognitive behavioral therapy
DDS: Diabetes Distress Scale
eMH: electronic mental health
GAD-7: Generalized Anxiety Disorder Questionnaire-7
GP: general practitioner
HbA_1c_: glycosylated hemoglobin
HREC: Human Research Ethics Committee
MMRM: mixed model repeated measure
NHMRC: National Health and Medical Research Council
PHQ-9: Patient Health Questionnaire-9 item
RCT: randomized controlled trial
SMP-T2D: Self-Management Profile for Type 2 Diabetes
T2DM: type 2 diabetes
WSAS: Work and Social Adjustment Scale
